# Changes in the Metabolome of *Picea balfouriana* Embryogenic Tissues That Were Linked to Different Levels of 6-BAP by Gas Chromatography-Mass Spectrometry Approach

**DOI:** 10.1371/journal.pone.0141841

**Published:** 2015-10-30

**Authors:** Q. F. Li, J. H. Wang, P. Pulkkinen, L. S. Kong

**Affiliations:** 1 State Key Laboratory of Forest Genetics and Tree Breeding, Research Institute of Forestry, Chinese Academy of Forestry, Beijing, China; 2 Guangxi Key Laboratory of Special Non-wood Forest Cultivation & Utilization, GuangXi Zhuang Autonomous Region Forestry Research Institute, Nanning, China; 3 Finnish Forest Research Institute, Haapastensyrjä, Finland; 4 Unit Centre for Forest Biology, Department of Biology, University of Victoria, Victoria, Canada; CSIR- Indian Institute of Toxicology Research, INDIA

## Abstract

Embryogenic cultures of *Picea balfouriana*, which is an important commercial species for reforestation in Southern China, easily lose their embryogenic ability during long-term culture. Embryogenic tissue that proliferated at lower concentrations (3.6 μM and 2.5 μM) of 6-benzylaminopurine (6-BAP) were more productive, and generated 113 ± 6 and 89 ± 3 mature embryos per 100 mg embryogenic tissue, respectively. A metabolomic approach was used to study the changes in metabolites linked to embryogenic competence related to three different 6-BAP concentrations (2.5 μM, 3.6 μM, and 5 μM). A total of 309 compounds were obtained, among which 123 metabolites mapped to Kyoto Encyclopedia of Genes and genomes (KEGG) pathways. The levels of 35 metabolites were significantly differentially regulated among the three 6-BAP treatments, and 32 metabolites differed between the 2.5 μM and 5 μM treatments. A total of 17 metabolites appeared only once among the three comparisons. The combination of a score plot and a loading plot showed that in the samples with higher embryogenic ability (3.6 μM and 2.5 μM), up-regulated metabolites were mostly amino acids and down-regulated metabolites were mostly primary carbohydrates (especially sugars). These results suggested that 6-BAP may influence embryogenic competence by nitrogen metabolism, which could cause an increase in amino acid levels and higher amounts of aspartate, isoleucine, and leucine in tissues with higher embryogenic ability. Furthermore, we speculated that 6-BAP may affect the amount of tryptophan in tissues, which would change the indole-3-acetic acid levels and influence the embryogenic ability.

## Introduction

The progressively diminishing embryogenic ability of embryogenic tissues has been well characterized in conifer trees. A primary example of such recalcitrance in spruce species is the inability or decreased competence of established embryogenic tissue to generate early stage embryos in response to suitable maturation conditions and to develop fully mature embryos. Although technology for the initiation and proliferation of somatic tissues and subsequent generation of mature cotyledonary embryos in spruces and other conifers has improved [1–10], some embryogenic tissues from many conifer species continue to exhibit a high degree of variability and others have lost embryogenic ability.

*Picea balfouriana* is an evergreen spruce tree that is distributed mostly in the southwest and northern regions of the Tibetan plateau. Because of the high quality of the wood and its fast growth, *P*. *balfouriana* is a major species of choice for afforestation. However, there are several drawbacks to using *P*. *balfouriana* for afforestation, including the fact that it reproduces primarily sexually, its seedlings initially grow slowly, and it sets seed late [11]. In our laboratory we have established the whole somatic embryogenesis system of *P*. *balfouriana* and applied for a patent [12]. We found that the embryogenic ability of the system was easily decreased when the amount of 2,4-dichlorophenoxyacetic acid (2,4-D) and 6-benzylaminopurine (6-BAP) in proliferation stage were maintained or removed, and realized there was an urgent need to study the early stage of somatic embryogenesis [13]. We found that if the level of 2,4-D was decreased during proliferation, the embryogenic tissue would hardly generate somatic embryos. The amount of the cytokinin 6-BAP that added in the proliferation stage would influence the final yield of mature embryos from tissues, especially during long-term culture. It is well known that cytokinins play important roles in the control of cell division in plants, and the cytokinin signaling pathway has been studied recently [14,15]. However, the mechanism of action of 6-BAP in plant somatic embryogenesis is hardly known. In a previous study, we found that the influence of 6-BAP on embryogenic capacity was through relevant mRNAs and proteins [16] and, therefore, we inferred that 6-BAP may affect the corresponding metabolites.

Metabolomics, the global analysis of cellular metabolites, is a powerful tool based on functional proteomics that can be applied to gain insights into biological functions, which may be an effective approach for the functional characterization of genes, and may help in the description and elucidation of physiological responses in plants under different environmental conditions [17–27]. Gas chromatography-mass spectrometry (GC-MS) is generally performed using electron-impact quadrupole or time-of-flight mass spectrometry [28] and is one of the most developed analytical platforms for plant metabolite profiling [29]. Using GC-MS, it is possible to profile several hundred compounds belonging to diverse chemical classes, including sugars, organic acids, amino acids, sugar alcohols, aromatic amines, and fatty acids. For example, the regulation of developmental events has been elucidated at the metabolic level using metabolic profiling [30, 31] and different capabilities of embryogenic cell lines of *Pinus taeda* L. [32] and *Picea abies* (L.) [33] had been explained by a model based on the combined data for metabolic profiles. Further, the combination of GC-MS and OPLS-DA (orthogonal projections to latent structures discriminant analysis) [34–37] has been used to visualize and discriminate interesting metabolites.

In the present study, we investigated the metabolic profiles of embryonic tissues using three 6-BAP concentrations and identified important metabolites that were affected by 6-BAP and associated with embryogenic competence. The objective was to use the GC-MS approach to investigate changes in the metabolome that were linked to embryogenic ability, which was related to different levels of 6-BAP.

## Materials and Methods

### Plant material and sampling

**Fig 1:**
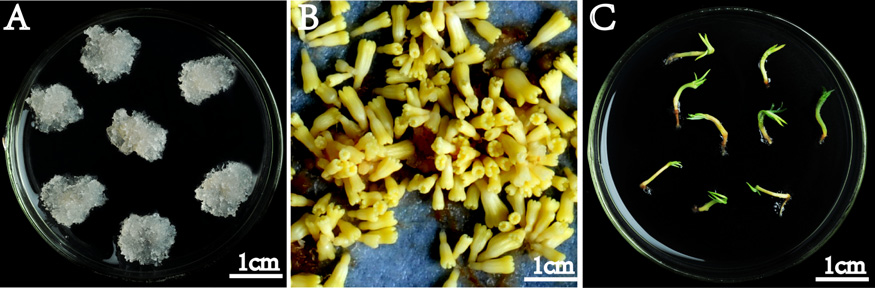
Somatic embryogenesis of *Picea balfouriana*. **(A)** Embryogenic tissues. **(B)** Somatic embryos. **(C)** Germination of somatic embryos.

One selected embryogenic cell line of *P*. *balfouriana* (Fig 1A) was used in this study. This cell line was established in 2011 and was initiated at the Research Institute of Forest, Chinese Academy of Forestry using seeds from elite genotype 4 that were induced on solidified half-strength LM medium [38] supplemented with 10 μM 2,4-D and 5 μM 6-BAP[12], 1% sucrose, 500 mg/L glutamine, 1 g/L casein hydrolysate, and 2% Gelrite at 24 ± 1°C in the dark. This cell line was proliferated on solidified half-strength LM medium with three concentrations of 6-BAP (2.5 μM, 3.6 μM, and 5 μM) and with other supplements kept unchanged at 24 ± 1°C in the dark. This produced embryogenic tissues with different embryogenic capabilities after 3 months. The embryogenic cultures were sub-cultured at 2-week intervals. This somatic embryogenesis culture experiment was performed twice. Both experimental series yielded similar results with respect to embryo development, and samples from one of the experimental series were selected for metabolomic profiling.

Samples of embryogenic culture were collected after subculturing for 7 days. For each treatment, six biological replicates were collected. The samples were denoted as 2.5 μM-, 3.6 μM-, and 5 μM-treated samples. All samples were transferred to cryotubes, flash frozen in liquid nitrogen, and stored at −80°C until further processing for metabolite extraction.

Early embryo differentiation from tissues was stimulated by transferring the cultures to half-strength LM medium lacking plant growth regulators for 1 week. Promotion of late embryo development and maturation (Fig 1B) was performed by transferring cultures to half-strength LM medium supplemented with 61 μM abscisic acid and 0.4% active charcoal, 6% sucrose, 500 mg/L glutamine, 1 g/L casein hydrolysate, and 4% Gelrite, and culturing at 24 ± 1°C in the dark. There were 10 replicates of each treatment. Then, somatic embryos (at least 3 mm long) generated from 100 mg embryogenic tissue that had 3–5 cotyledons and could germinate were counted after 8 weeks (Fig 1C). Twenty somatic embryos from each of the three 6-BAP treatments were put on one quarter-strength LM medium with 0.5% active charcoal, 2% sucrose, 500 mg/L glutamine, 500 mg/L casein hydrolysate, and 3% Gelrite for germination at 24 ± 1°C in the light (30 μEm^-2^s^-1^, 16 h photoperiod). There were 10 replicates for each treatment. After 6 weeks, the numbers of germinated somatic embryos with elongated root and hypocotyl were counted.

### Histological analysis

Another cell line that was induced from elite genotype 3 in 2013 and treated in the same way as the test group was used as the control to validate the influence of 6-BAP. The numbers of mature somatic embryos and germinated somatic embryos of the control line were counted in the same way as in the test group. Histological differences between the test and control lines were analyzed. Proliferated tissues on medium supplemented with different levels of 6-BAP were treated and stained for general light microscopy observations according to Gupta and Holmstrom [39] with some modification. The embryonal head cells were stained bright red (acetocarmine) and suspensor cells were stained blue. These two sections together were considered as the early stage embryos and the numbers of early stage embryos of each 6-BAP concentration were counted.

### Statistical analysis

To compare the influence of different levels of 6-BAP on the maturation of tissues and the germination of their generated somatic embryos, the numbers of early somatic embryos, the numbers of mature somatic embryos, and the germination rates were subjected to analysis of variance (ANOVA) using SPSS20 software(http://www-01.ibm.com/software/analytics/spss/downloads.html). The level of significance was P <0.05.

### Metabolite extraction and gas chromatography–time-of-flight mass spectrometry (GC/TOF/MS) analysis

Metabolites from embryogenic tissue (100-mg fresh weight) were extracted according to Lisec et al. [40] with minor modifications. Briefly, embryogenic tissue stored at −80°C was ground in a mortar using liquid nitrogen, and transferred into 2-mL centrifuge tubes. Ribitol (60 μL) was added and vortexed for 10 s, followed by the addition of 0.35 mL 100% methanol and vortexing for 10 s. The tubes were placed into an ultrasound machine at 70°C for 10 min, and then centrifuged for 10 min at 12,000 rpm at 4°C. Next, 0.35 mL supernatant was transferred into new Eppendorf tubes and samples were blow-dried using moderate nitrogen. Methoxamine hydrochloride (80 μL) was added, vortexed for 30 s, and allowed to react for 2 h at 37°C. Finally, 100 μL BSTFA reagent (containing 1% TMCS, v/v) was added to the mixture and allowed to react for 1 h at 70°C.

The GC/TOF/MS analysis was performed using an Agilent 7890A gas chromatograph system coupled with a Pegasus four-dimensional time-of-flight mass spectrometer (Agilent, USA). The system used a DB-5MS capillary column coated with 5% diphenyl cross-linked with 95% dimethylpolysiloxane (30 m × 250-μm inner diameter, 0.25 μm film thickness; J&W Scientific, Folsom, CA, USA). Next, a 1-μL aliquot of the analyte was injected in splitless mode. Helium was used as the carrier gas, the front inlet purge flow was 3 mL min^−1^, and the gas flow rate through the column was 1 mL min^−1^. The initial temperature was maintained at 90°C for 0.25 min, and then raised to 240°C at a rate of 5°C min^−1^, and finally to 285°C at a rate of 20°C min^−1^ for 11.5 min. The injection, transfer line, and ion source temperatures were 280°C, 250°C, and 220°C, respectively. The energy was −70 eV in electron-impact mode. The MS data were acquired in full-scan mode with the m/z range of 20–600 at a rate of 100 spectra per second after a solvent delay of 492 s.

Multivariate and statistical analyses of raw signals, data baseline filtering, and peak identification and integration were performed using the Simca software (http://www.umetrics.com/products/simca). The data were then imported into the TagFinder software [41] with default parameters for correction of retention time to mass debris, peak alignment, and deconvolution analysis [42]. The total mass of the signal integration area was normalized for each sample; that is, the total integral area of each sample was set as 1000. Then, principal component analysis (PCA) of internal standard peak areas was performed to provide sample weights and *t1* scores to normalize the data before multivariate analysis. In addition, metabolite data were mean centered and UV scaled. To obtain an overview of the metabolite data, a PCA model was calculated initially on the X-matrix [43]. In the PCA, a few latent variables were calculated, which described the largest systematic variation in the X-matrix. Thus, both the influence of noise and dimensionality on the data was greatly reduced. Using the resultant PCA score scatters, clusters and outliers within samples can be identified [44]. Finally, the normalized data were imported into SIMCA-P + 12.0.1 (Umetrics AB, Umeå, Sweden) using the OPLS-DA model with the first principal component of VIP (variable importance in the projection) values (VIP >1) combined with the Student’s t-test (t-test) (P <0.05) to identify differentially expressed metabolites and to search for metabolites in commercial databases such as those provided by the National Institute of Standards and Technology (NIST; http://www.nist.gov/index.html) and the publicly available KEGG (Kyoto Encyclopedia of Genes and Genomes) database (http://www.genome.jp/kegg/). To characterize the physiological mechanisms of early somatic embryogenesis underlying the effects of the 6-BAP treatments, we examined the metabolic changes in embryogenic tissue at three 6-BAP concentrations. For each treatment, three comparisons were made: 2.5 μM *vs* 5 μM, 3.6 μM *vs* 2.5 μM, and 3.6 μM *vs* 5 μM. In each comparison, the sample with the lower embryo production was always made the control group. Metabolites that differed between samples were identified using OPLS-DA loading plots and a t-test of the respective metabolite peak areas. In all cases, models were judged for quality using goodness of fit (R^2^X) and goodness of prediction parameters.

### Visualization

If interesting metabolites are selected based solely on the correlation, a number of biochemical compounds that are present in very low concentrations also will be selected, and the risk of selecting false positives will be high. Potentially biochemically interesting compounds can be better selected based on a combination of covariance and correlation information, which is the purpose of the score plot (S-plot). The farther along the x-axis (covariance), the greater the contribution to the variance between the groups, while the farther the y-axis (correlation), the higher the reliability of the analytical result.

OPLS-DA together with the S-plot and loading plot allows complex data to be mined for metabolites that are statistically and potentially biochemically interesting compounds. The use of appropriate visualization tools helps in the communication and interpretation of scientific data. The metabolic data were analyzed as described by Wiklund *et al*. [36]. Briefly, the two vectors used in the S-plot are calculated as

$$Cov(t,Xi)=\frac{t^{T}X_{i}}{N-1}$$ (1)

$$Corr(t,Xi)=\frac{Cov(t,Xi)}{s_{t}s_{X_{i}}}$$ (2)

where *t* is the score vector in the OPLS-DA model, *i* is the centered variable in data matrix *X*, and *s* is the estimated standard deviation. Therefore, *Cov*(*t*,*X*) and *Corr*(*t*,*X*) are vectors with the same length as the number of variables in the mode. These vectors are plotted in a scatter plot and are S-shaped unless the variable variance is uniform. The x-axis (*Cov*(*t*,*X*)) in the S-plot is a visualization of contribution (covariance), and the y-axis (*Corr*(*t*,*X*)) spans a theoretical minimum (−1) and maximum (+1), where 1 is the correlation (reliability). The statistical S-plot was used to identify possible biochemically interesting compounds for both the predictive and orthogonal variations. A complementary tool for identifying interesting compounds is the loading plot where the vector *Cov*(*tp*,*X*), which includes the corresponding jack-knifed confidence intervals, provides additional information about metabolite variability.

## Results

### Development of *P*. *balfouriana* somatic embryos

During the maturation stage, embryogenic tissue of the cell lines exhibited different embryogenic ability after being treated with 2.5 μM, 3.6 μM, or 5 μM 6-BAP. Embryogenic tissue proliferated in medium containing 3.6 μM 6-BAP and yielded the most fully mature somatic embryos with a normal set of cotyledons (96±7/per 100 mg tissue), which had higher germination rates (48.47%±0.06)) than the mature somatic embryos from the other two 6-BAP treatments. Medium containing 2.5 μM 6-BAP yielded the second highest number of mature embryos (64±4/per 100 mg tissue) with germination rates of 28.39±0.04 and medium containing 5 μM 6-BAP yielded the lowest number of mature somatic embryos (11±1/per 100 mg tissue) with the lowest germination rates (9.42%±0.03).

**Fig 2:**
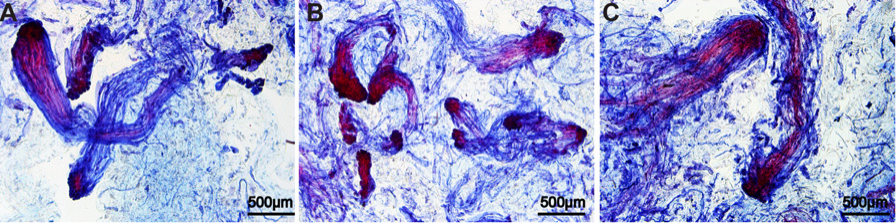
Early stage embryos in tissues treated with 6-BAP by micro-examination. The bars in each of the panels indicate 500 μm. **(A)** Early somatic embryos in tissue treated with 2.5 μM 6-BAP. **(B)** Early somatic embryos in tissue treated with 3.6 μM 6-BAP. **(C)** Early and mature somatic embryos in tissue treated with 5 μM 6-BAP.

ANOVA of the control line also revealed significant differences among the samples treated with the three concentrations of 6-BAP (Table 1). In addition, a micro-examination showed that the numbers of early somatic embryos in the different treatments were significantly different (Fig 2); that is, 31±3, 47±4, and 5±2 early somatic embryos per 50 mg tissue in the 2.5 μM, 3.6 μM, and 5 μM groups, respectively.

**Table 1:** Number of mature cotyledonary embryos generated from each treatment (per 100 mg of embryogenic tissue) and their germination rates compared with the control line.

Sample	Mature embryos / per 100 mg of embryogenic tissue	Germination rate
**2.5 μM**	64 ± 4^b^	28.39% ± 0.04^b^
**3.6 μM**	96 ± 7^a^	48.47% ± 0.06^a^
**5 μM**	11 ± 1^c^	9.42% ± 0.03^c^

^a, b, c^ indicate the significance of difference (P ≤0.05).

### Metabolic changes in response to 6-BAP

**Fig 3:**
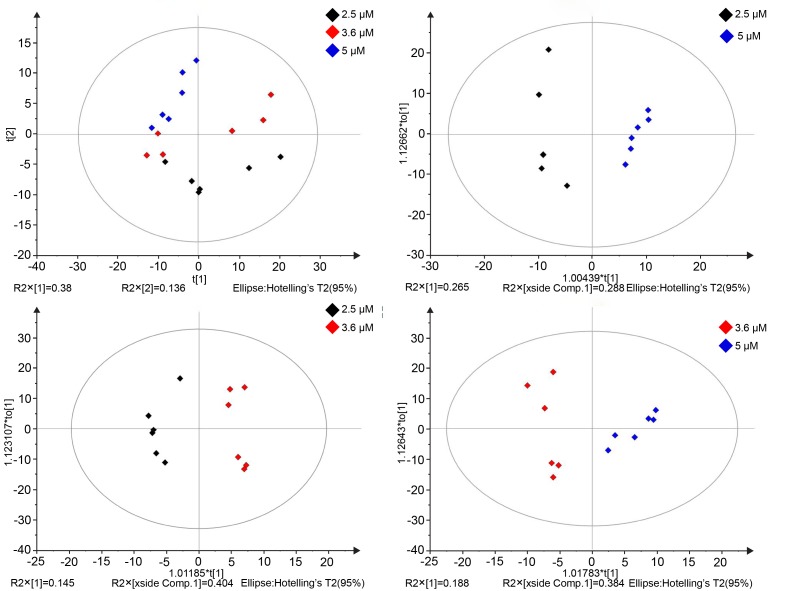
Score plots for the first two principal components by multivariate analysis. **(A)** PCA *t1*/*t2* score scatter (N = 18, R^2^X [1] = 0.38, R^2^X [2] = 0.14, and Q^2^Y [cum] = 0.36). OPLS-DA score scatters. **(B)** 2.5 μM versus 5 μM (R^2^X = 0.553, R^2^Y = 0.96 and Q^2^ = 0.834). **(C)** 3.6 μM versus 2.5 μM (R^2^X = 0.548, R^2^Y = 0.95 and Q^2^ = 0.742). **(D)** 3.6 μM versus 5 μM (R2X = 0.573, R2Y = 0.89 and Q2 = 0.676). Samples are colored according to the 6-BAP concentrations.

Analysis of the three 6-BAP-treated culture samples yielded 309 compounds, among which 123 metabolites were assigned to KEGG pathways. A PCA model with two principal components explained 52% of the variation across the three samples (Fig 3A).

S-plot for the first two principal components of each two samples (2.5 μM versus 5 μM, 3.6 μM versus 2.5 μM and 3.6 μM versus 5 μM), explained 54%, 59% and 58% of the total variation, respectively, and was used for an overview of the data. The S-plot showed that each 6-BAP-treated sample was separated from the main cluster and distributed. OPLS-DA was used to discriminate between the two samples, and the OPLS-DA showed that the samples were separated according to the 6-BAP concentration (Fig 3B, 3C and 3D). Metabolites that distinguished the sample classes are presented in S1 Table (2.5 μM versus 5 μM), S2 Table (3.6 μM versus 2.5 μM) and S3 Table (3.6 μM versus 5 μM), respectively.

### 2.5 μM versus 5 μM 6-BAP-treated samples

Most of the differentially regulated metabolites (32) were present in the 2.5 μM group compared with the 5 μM group, both of which had lower embryogenic competence than the 3.6 μM group. The majority of the regulated metabolites (14 up-regulated, four down-regulated) were amino acids or their derivatives associated with various biosynthetic pathways. Among the regulated metabolites that were carbohydrates, the number of up- and down-regulated metabolites was approximately equal. A total of eight organic acids (five up-regulated, three down-regulated) were identified in the 2.5 μM group compared with the 5 μM group; notably, lyxose 1 was significantly increased (19-fold) in the 5 μM 6-BAP group.

**Fig 4:**
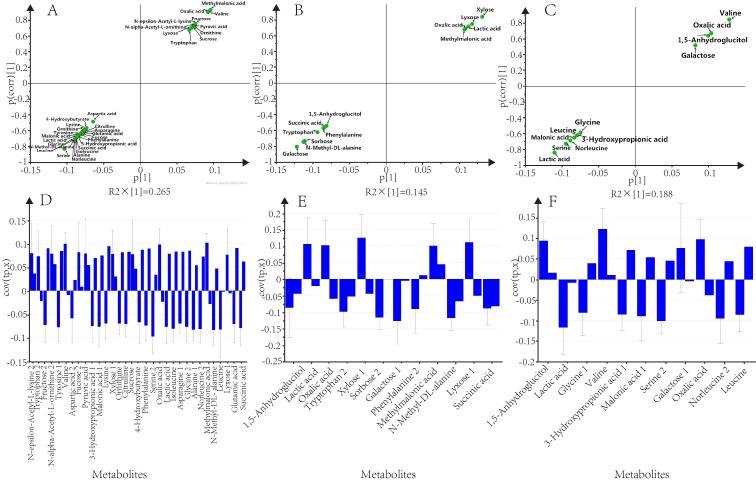
Strategy for identification of interesting metabolites. **(A)**, **(B)**, and **(C)** show selected significant metabolites related to the differences between each pairwise comparison (see Fig 3). **(D)**, **(E)**, and **(F)** show the loading plots derived from each pairwise comparison. The plots mainly show the selected metabolites from the S-plot (see Fig 3).

The separation between the 32 differentially regulated metabolites between the 2.5 μM and 5 μM groups was highlighted in the S-plot (Fig 4A). In the raw data plot both groups overlapped, while only 11 metabolites had a high correlation in the S-plot and a high reliability in the loading plot (Fig 4D) and were deemed reliable for separating the groups.

### 3.6 μM versus 2.5 μM 6-BAP-treated samples

Three differentially regulated metabolites were up-regulated and two were down-regulated in the 3.6 μM group compared with the 2.5 μM group, and three were amino acids, of which one was down-regulated and two were up-regulated in the 2.5 μM group compared with the control cells. Among the regulated metabolites that were organic acids, the number of up- and down-regulated metabolites was equal.

In the S-plot, 12 metabolites showed a high correlation (Fig 4B); however, in the loading plot, most of them showed low reliability because the confidence interval did not support the S-plot selection (Fig 4E). Finally, only tryptophan 2 and sorbose 2 showed reasonable reliability were selected for further investigation of their biochemical significance.

### 3.6 μM versus 5 μM 6-BAP-treated samples

Three of the differentially regulated metabolites were carbohydrates; one was up-regulated and two were down-regulated in 3.6 μM cultures compared with 5 μM. A common pattern was observed for the organic acid and amino acid groups, both of which comprised four compounds with one down-regulated and three up-regulated in the 5 μM culture compared with the control cells. The differentially regulated metabolites with the highest levels in the 3.6 μM group were oxalic acid, galactose and, leucine, among which the leucine level was significantly up-regulated compared with the 5 μM group.

Significant differences between the 3.6 μM and 5 μM samples were identified using the mass peak intensities of all the detected metabolites, which were expressed in an S-plot, OPLS-DA (Fig 4C, and loading plot (Fig 4F). Both up-regulated and down-regulated metabolites were found in the 3.6 μM samples compared with 5 μM samples, but only 1,5-anhydroglucitol, 3-hydroxypropionic acid 1 and leucine were selected as significant regulated markers based on the statistical analyses.

## Discussion

In the present study, we followed the metabolic events in one embryogenic cell line of *P*. *balfouriana* that displayed different embryogenic activities after being treated with three 6-BAP concentrations. Of the embryogenic tissues, those treated with 3.6 μM 6-BAP generated the greatest number of somatic embryos, which also had higher germination rates than the somatic embryos generated from embryogenic tissues proliferated on medium with lower (2.5 μM) or higher (5 μM) levels of 6-BAP. Furthermore, micro-examination showed that the embryonal heads and suspensors of early somatic embryos in the 5 μM-treated cultures were bigger than those in the 2.5 μM- and 3.6 μM-treated cultures. These results together with the ANOVA of mature embryos and histological analysis of early somatic embryos in the control cell line demonstrated that 6-BAP influenced the maturation of tissues and showed that the influence had universality. Many possible mechanisms could be proposed to explain these results; for example, 6-BAP may have influenced other plant growth regulators or genes involved in metabolite regulation. In the present study, we investigated this phenomenon using a metabolomics approach and found that metabolite profiles were altered significantly in response to different concentrations of 6-BAP. The preferential differential regulation of metabolites may trigger adaptive responses during somatic embryogenesis.

Although the t-test is widely used for selecting interesting compounds, we found that some of the regulated metabolites identified using the t-test may not be reliable [45–47]. For example, lyxose1 was significantly up-regulated (19-fold) in the 5 μM 6-BAP group compared with the 2.5 μM group and was selected as a reliably regulated metabolite based on the loading plot, but would not have been selected based on the t-test. The major dissent is that the t-test gives no consideration to variable intensity, which is often related to metabolite concentration [48]. In the present study, we used a loading plot because it reduces the impact of artifacts and noise in the models.

Leucine was up-regulated in embryogenic tissues proliferated in the 3.6 μM culture compared with the 5 μM culture, while sorbose 2 was significantly down-regulated in the 3.6 μM culture compared with the 2.5 μM culture. The aspartic acid, alanine, asparagine, serine, glycine, and phenylalanine amino acids were up-regulated in the 2.5 μM culture compared with the 5 μM culture, which did not efficiently generate embryos. This finding is in good agreement with the results of Richard et al. [49]. Broeckling et al. [50] reported a negative correlation between amino acid and sugar levels in embryogenic tissues, which is in agreement with our results. We found that amino acid metabolites were mostly up-regulated, whereas most of the sugars were down-regulated. All the metabolites that were differentially regulated by 6-BAP resulted in a different embryogenic capacity of the cultures.

6-BAP may affect two channels involved in early somatic embryogenesis: nitrogen metabolism and/or the IAA concentration in tissues. In nitrogen metabolism, NH_4_^+^ and NO_3_^−^ are both essential for the proliferation of embryogenic tissue and development of somatic embryos [51–53]. Ikram and Yusuf [54] found that when both KNO_3_ and NH_4_NO_3_ were added to the embryo-induction medium, there was a considerable increase in the rates of cell growth and somatic embryogenesis. We found that the asparagine level was higher in the 2.5 μM group compared with the 5 μM group. Asparagine was shown to be a key component of nitrogen metabolism in conifers [55]. Furthermore, isoleucine and norleucine levels were also higher in the 2.5 μM group compared with the 5 μM group, and these two amino acids are synthesized by aspartate. Aspartate is synthesized from glutamate in the plastid and channeled through the aspartate metabolic pathway for the biosynthesis of lysine, threonine, isoleucine, methionine, and other essential nitrogen compounds [56]. Taken together, our data suggest that 2.5 μM 6-BAP increased the levels of several amino acids that are involved in nitrogen metabolism, which is indispensable for the proliferation of embryogenic cultures.

Another channel that we propose could be involved in early somatic embryogenesis may be affected by the influence of 6-BAP on the amount of IAA in tissues. The tryptophan level was lowest in cultures treated with 3.6 μM 6-BAP. Tryptophan can be converted to indole-3-pyruvic acid, which is then converted to indole-3-acetaldehyde from which IAA is synthesized [57]. IAA can also be synthesized by converting tryptophan to indole-3-acetaldoxime, which is then converted to indole-3-acetonitrile from which IAA is synthesized [33]. Furthermore, the interaction between auxin and cytokinin has been found to control cell proliferation and differentiation [58–60]. Therefore, our results indicated that the embryogenic ability of *P*. *balfouriana* embryogenic tissue could be maintained during the long proliferation stage of somatic embryogenesis by adding moderate concentrations of 6-BAP to the media. This technique for somatic embryogenesis could be applied to promote afforestation by *P*. *balfouriana*. In addition, the differentially regulated metabolites (asparagine, tryptophan, and others) may be used as markers to detect the embryogenic competence of tissues.

## Supporting Information

S1 Table Levels of regulated metabolites in groups 2.5 μM and 5 μM (P<0.05). (DOCX)

S2 Table Levels of regulated metabolites in groups 2.5 μM and 3.6 μM (P < 0.05). (DOCX)

S3 Table Metabolites differentially regulated in 3.6 μM and 5 μM (P < 0.05). (DOCX)
