# Supplementary material for: Changes in the Metabolome of Picea balfouriana Embryogenic Tissues That Were Linked to Different Levels of 6-BAP by Gas Chromatography-Mass Spectrometry Approach
Source: PLoS One. 2015 Oct 30;10(10):e0141841. doi: 10.1371/journal.pone.0141841 (PMC4627733; doi:10.1371/journal.pone.0141841)
Supplement: S1 Table — (DOCX) [file pone.0141841.s001.docx]

**S1 Table. Levels of regulated metabolites in groups 2.5 μM and 5 μM (P<0.05)**

| Metabolite class | Metabolite name | RT | Similarity | VIP | P  value | Fold change |
| --- | --- | --- | --- | --- | --- | --- |
| Organic Acids and Derivatives | Pyruvic acid | 6.185 | 826 | 1.448 | 0.007 | -0.285 |
|  | Malonic acid 1 | 26.767 | 875 | 1.308 | 0.020 | 1.931 |
|  | Oxalic acid | 7.060 | 708 | 1.712 | 0.000 | -1.236 |
|  | Methylmalonic acid | 7.147 | 762 | 1.675 | 0.001 | -2.620 |
|  | 3-Hydroxypropionic  acid 1 | 7.255 | 858 | 1.275 | 0.025 | 1.216 |
|  | 4-Hydroxybutyrate | 8.359 | 920 | 1.186 | 0.042 | 1.986 |
|  | Lactic acid | 6.297 | 969 | 1.261 | 0.027 | 0.599 |
|  | Succinic acid | 23.943 | 952 | 1.362 | 0.014 | 1.284 |
| Carbohydrates and Carbohydrate Conjugates | Glycine 2 | 9.289 | 981 | 1.277 | 0.025 | 1.169 |
|  | Lyxose 1 | 16.729 | 837 | 1.434 | 0.008 | -19.410 |
|  | Xylose 1 | 13.767 | 886 | 1.393 | 0.011 | -0.876 |
|  | Fructose 2 | 16.339 | 805 | 1.395 | 0.011 | -2.794 |
|  | Sucrose | 25.485 | 908 | 1.306 | 0.021 | -1.052 |
|  | Fucose 2 | 23.609 | 728 | 1.270 | 0.026 | 1.648 |
|  | Tyrosine 1 | 18.349 | 769 | 1.375 | 0.013 | 1.556 |
| Amino Acids, Peptides, and Analogues | Tryptophan 2 | 10.839 | 719 | 1.315 | 0.020 | -0.663 |
|  | Serine 2 | 8.651 | 943 | 1.749 | 0.000 | 1.765 |
|  | Alanine 1 | 6.828 | 990 | 1.343 | 0.016 | 1.130 |
|  | Isoleucine | 9.077 | 969 | 1.391 | 0.013 | 1.255 |
|  | Aspartic acid 2 | 10.731 | 805 | 1.157 | 0.048 | 1.428 |
|  | Leucine | 10.533 | 935 | 1.378 | 0.013 | 16.639 |
|  | Glutamic acid | 23.094 | 854 | 1.201 | 0.038 | 2.383 |
|  | N-alpha-Acetyl-L-ornithine 2 | 13.048 | 768 | 1.242 | 0.030 | -0.395 |
|  | Asparagine 1 | 14.083 | 970 | 1.166 | 0.046 | 1.377 |
|  | N-epsilon-Acetyl-L-lysine 2 | 9.529 | 713 | 1.224 | 0.034 | -0.558 |
|  | Lysine | 18.077 | 880 | 1.198 | 0.039 | 1.199 |
|  | Valine | 5.518 | 780 | 1.684 | 0.001 | -2.213 |
|  | Norleucine 2 | 7.763 | 873 | 1.414 | 0.010 | 2.215 |
|  | Ornithine | 13.282 | 891 | 1.173 | 0.044 | 1.691 |
|  | N-Methyl-DL-  alanine | 7.993 | 936 | 1.468 | 0.006 | 0.892 |
|  | Phenylalanine 1 | 13.575 | 924 | 1.269 | 0.026 | 1.291 |
|  | Citrulline 1 | 16.475 | 905 | 1.223 | 0.034 | 1.110 |
