# Supplementary material for: Changes in the Metabolome of Picea balfouriana Embryogenic Tissues That Were Linked to Different Levels of 6-BAP by Gas Chromatography-Mass Spectrometry Approach
Source: PLoS One. 2015 Oct 30;10(10):e0141841. doi: 10.1371/journal.pone.0141841 (PMC4627733; doi:10.1371/journal.pone.0141841)
Supplement: S2 Table — (DOCX) [file pone.0141841.s002.docx]

**S2 Table. Levels of regulated metabolites in groups 2.5 μM and 3.6 μM (P < 0.05)**

| Metabolite class | Metabolite name | RT | Similarity | VIP | P  value | Fold change |
| --- | --- | --- | --- | --- | --- | --- |
| Organic Acids and Derivatives | Lactic acid | 14.272 | 969 | 2.003 | 0.007 | -3.791 |
|  | Succinic acid | 23.943 | 952 | 1.592 | 0.046 | -1.308 |
|  | Methylmalonic acid | 7.916 | 7 62 | 1.782 | 0.021 | 0.657 |
|  | Oxalic acid | 6.630 | 708 | 1.698 | 0.031 | 1.023 |
| Carbohydrates and Carbohydrate Conjugates | Xylose 1 | 13.302 | 886 | 2.050 | 0.005 | 1.005 |
|  | 1,5-Anhydroglucitol | 13.683 | 715 | 1.588 | 0.047 | -1.368 |
|  | Lyxose 1 | 14.107 | 837 | 1.927 | 0.010 | 0.837 |
|  | Galactose 1 | 17.369 | 839 | 2.373 | 0.000 | -2.329 |
|  | Sorbose 2 | 13.767 | 887 | 1.972 | 0.008 | -0.614 |
| Amino Acids, Peptides, and Analogues | Tryptophan 2 | 22.166 | 719 | 1.764 | 0.023 | -2.290 |
|  | N-Methyl-DL-alanine | 7.993 | 936 | 2.084 | 0.004 | 3.831 |
|  | Phenylalanine 2 | 12.541 | 966 | 1.873 | 0.014 | 1.660 |
