# Supplementary material for: Changes in the Metabolome of Picea balfouriana Embryogenic Tissues That Were Linked to Different Levels of 6-BAP by Gas Chromatography-Mass Spectrometry Approach
Source: PLoS One. 2015 Oct 30;10(10):e0141841. doi: 10.1371/journal.pone.0141841 (PMC4627733; doi:10.1371/journal.pone.0141841)
Supplement: S3 Table — (DOCX) [file pone.0141841.s003.docx]

**S3 Table. Metabolites differentially regulated in 3.6 μM and 5 μM (P < 0.05)**

| Metabolite class | Metabolite name | RT | Similarity | VIP | P  value | Fold change |
| --- | --- | --- | --- | --- | --- | --- |
| Organic Acids and Derivatives | Oxalic acid | 7.060 | 708 | 1.649 | 0.018 | 0.786 |
|  | 3-Hydroxypropionic acid 1 | 7.255 | 858 | 1.478 | 0.041 | 1.470 |
|  | Malonic acid 1 | 26.767 | 875 | 1.669 | 0.016 | 1.682 |
|  | Lactic acid | 14.272 | 969 | 2.125 | 0.000 | 0.753 |
| Carbohydrates and Carbohydrate Conjugates | 1,5-Anhydroglucitol | 13.302 | 715 | 1.458 | 0.044 | -0.477 |
|  | Galactose 1 | 17.659 | 839 | 1.549 | 0.030 | -1.882 |
|  | Glycine 1 | 7.009 | 776 | 1.443 | 0.047 | 2.389 |
| Amino Acids, Peptides, and Analogues | Valine | 5.518 | 780 | 2.121 | 0.000 | -2.108 |
|  | Norleucine 2 | 7.478 | 873 | 1.452 | 0.045 | 2.646 |
|  | Serine 2 | 8.651 | 943 | 1.928 | 0.003 | 1.238 |
|  | Leucine | 10.533 | 935 | 1.429 | 0.049 | 16.297 |
